# Exploring the therapeutic mechanisms and prognostic targets of Biochanin A in glioblastoma via integrated computational analysis and in vitro experiments

**DOI:** 10.1038/s41598-024-53442-0

**Published:** 2024-02-15

**Authors:** Wanwen Ge, Guoqiang Yuan, Dongping Wang, Li Dong

**Affiliations:** 1 Lanzhou University Second Hospital, Lanzhou, 730030 China; 2 Gansu Provincial Hospital, Lanzhou, 730000 China; 3 Gansu University of Chinese Medicine, Lanzhou, 730000 China

**Keywords:** Medical research, Neurology

## Abstract

Glioblastoma (GBM) is the most aggressive brain tumor and is characterized by a poor prognosis and high recurrence and mortality rates. Biochanin A (BCA) exhibits promising clinical anti-tumor effects. In this study, we aimed to explore the pharmacological mechanisms by which BCA acts against GBM. Network pharmacology was employed to identify overlapping target genes between BCA and GBM. Differentially expressed genes from the Gene Expression Profiling Interactive Analysis 2 (GEPIA2) database were visualized using VolcaNose. Interactions among these overlapping genes were analyzed using the Search Tool for the Retrieval of Interacting Genes/Proteins database. Protein–protein interaction networks were constructed using Cytoscape 3.8.1. The Kyoto Encyclopedia of Genes and Genomes pathway and Gene Ontology enrichment analyses were conducted using the Database for Annotation, Visualization, and Integrated Discovery. Survival analyses for these genes were performed using the GEPIA2 database. The Chinese Glioma Genome Atlas database was used to study the correlations between key prognostic genes. Molecular docking was confirmed using the DockThor database and visualized with PyMol software. Cell viability was assessed via the CCK-8 assay, apoptosis and the cell cycle stages were examined using flow cytometry, and protein expression was detected using western blotting. In all, 63 genes were initially identified as potential targets for BCA in treating GBM. Enrichment analysis suggested that the pharmacological mechanisms of BCA primarily involved cell cycle inhibition, induction of cell apoptosis, and immune regulation. Based on these findings, *AKT1*, *EGFR*, *CASP3*, and *MMP9* were preliminarily predicted as key prognostic target genes for BCA in GBM treatment. Furthermore, molecular docking analysis suggested stable binding of BCA to the target protein. In vitro experiments revealed the efficacy of BCA in inhibiting GBM, with an IC50 value of 98.37 ± 2.21 μM. BCA inhibited cell proliferation, induced cell apoptosis, and arrested the cell cycle of GBM cells. Furthermore, the anti-tumor effects of BCA on U251 cells were linked to the regulation of the target protein. We utilized integrated bioinformatics analyses to predict targets and confirmed through experiments that BCA possesses remarkable anti-tumor activities. We present a novel approach for multi-target treatment of GBM using BCA.

## Introduction

Glioblastoma (GBM) is a prevalent primary malignant carcinoma of the central nervous system, known for its high mortality rate. Patients diagnosed with GBM typically have a less than 2-year median survival^1^. This condition is characterized by rapid growth, aggressive progression, and frequent relapse^2^. The current clinical treatment for GBM primarily involves surgical intervention combined with radiochemotherapy. Owing to its high blood–brain barrier permeability, temozolomide is the most commonly used drug for GBM treatment. However, challenges such as chemotherapeutic resistance and genotype insensitivity have significantly reduced the 5-year survival rate for malignant GBM to a mere 6.6%. Various mechanisms, such as point mutations and alterations in the tumor microenvironment, have led to GBM diversity and drug resistance.

Traditional Chinese herbs and natural products have been utilized for over 2000 years as anticancer agents in the treatment of various tumors, including gliomas^3^. Traditional Chinese medicine is effective in preventing and treating GBM, being primarily beneficial in alleviating toxic side effects, enhancing the effects of radiotherapy and chemotherapy, improving quality of life, reducing recurrence rates, and extending patient survival. Biochanin A (BCA), an isoflavone found in peanuts, cabbage, and red clover, has multi-targeted and diverse bioactive effects, including anti-tumor, anti-inflammatory, antioxidant, neuroprotective, and hepatoprotective properties. BCA primarily modulates the MAPK, PI3K, NRF2, and NF-κB signaling pathways to exert its bioactive effects^4^. Recent studies show that BCA hinders tumor growth by inducing cell apoptosis, inhibiting metastasis, and causing cell cycle arrest^5^. Furthermore, BCA regulates the expression of BGLAP, BAX, and ATF3, thereby suppressing osteosarcoma proliferation^6^.

**Figure 1:**
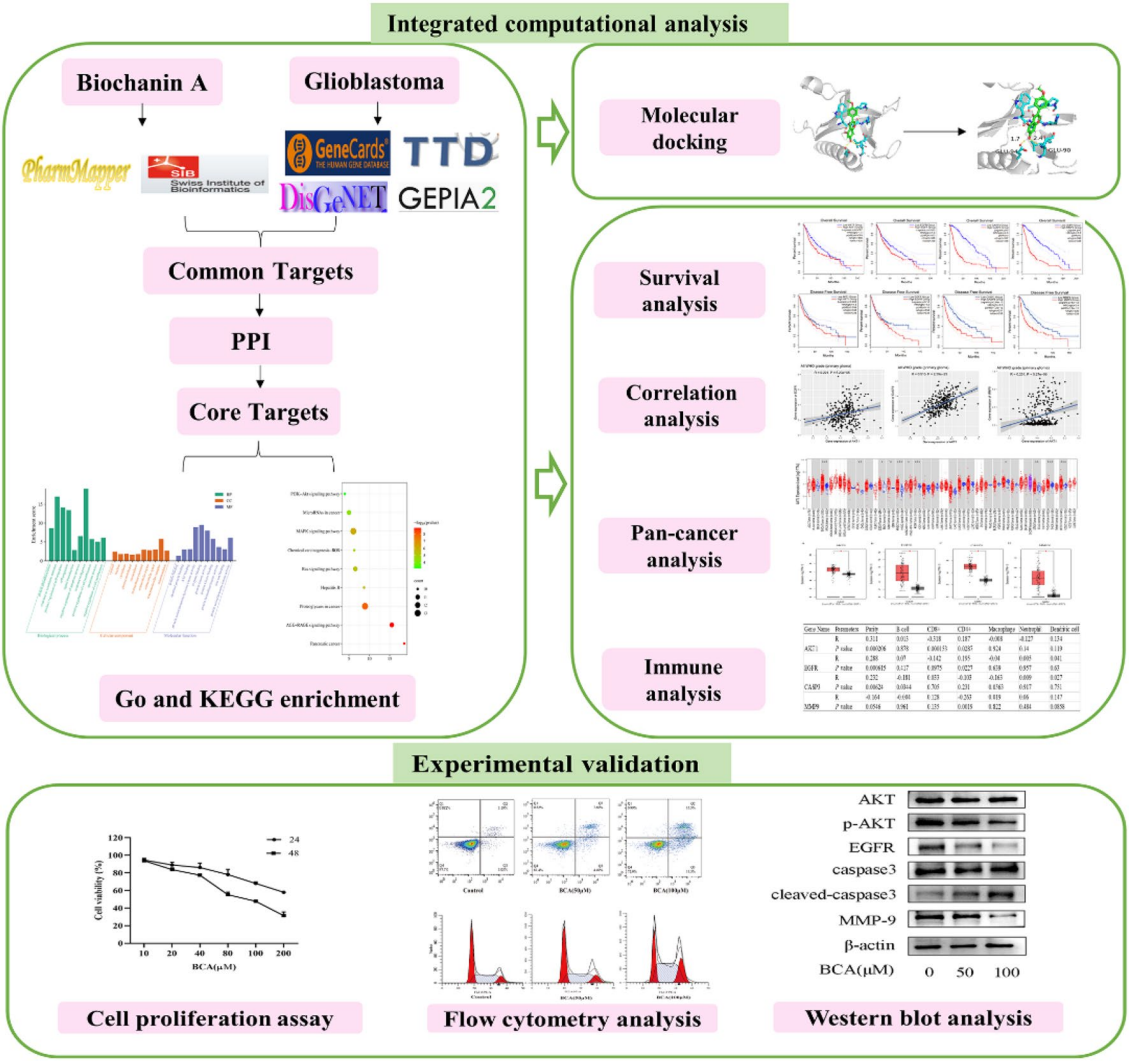
Flow chart depicting the research process.

Network pharmacology is emerging as a promising and cost-effective methodology for research and analysis. It identifies potential target genes for diseases and drugs by integrating systems biology and polypharmacology. Previous research involving tumor bioinformatics analysis has helped identify differentially expressed genes (DEGs) from the Gene Expression Profiling Interactive Analysis 2 (GEPIA2) database. This approach, which is based on standardized analysis of sequencing data, has provided new target genes for molecular mechanism research^7^. Furthermore, molecular docking is widely used for predicting interactions between target proteins and small molecular ligands^8^. In this study, we combined network pharmacology, bioinformatic analyses, and molecular docking to develop a comprehensive and multifaceted approach to investigate the effect of BCA on GBM. In addition, we performed in vitro experiments to validate the central genes and mechanisms involved in BCA treatment of GBM, paving the way for novel future clinical treatments. The detailed framework of the study is shown in Fig. 1.

## Material and methods

### Screening candidate targets of BCA and GBM

The SMILES and 3D structures of BCA were downloaded from PubChem (https://pubchem.ncbi.nlm.nih.gov/). The targets of BCA were sourced from the PharmMapper (http://lilab-ecust.cn/pharmmapper/submitfile.html)^9^ and Swiss Target Prediction (http://www.swisstargetprediction.ch/) databases. Duplicate entries were removed. GBM targets were sourced from three online databases: the Therapeutic Target Database (TTD, https://db.idrblab.net/ttd/), DisGeNet database (https://www.disgenet.org/), and GeneCards database (http://www.genecards.org/). The UniProt database (https://www.uniprot.org/) was used to standardize each gene. Consequently, BCA-associated targets were aligned with the targets related to GBM to identify potential targets of BCA against GBM.

### Screening of DEGs and hub genes

We obtained DEGs by comparing GBM and healthy controls using the GEPIA2 (http://gepia2.cancer-pku.cn/#index) database, the Genotype-Tissue Expression (GTEx) and The Cancer Genome Atlas (TCGA) projects. The dataset was set to include both GBM and low-grade glioma samples. DEGs were identified based on the following criteria: |Fold Change(log2)|> 1 and an adjusted *p* < 0.05. A volcano map was created using VolcaNoseR^10^ based on the following criteria: |Fold Change(log2)|> 1.5 and an adjusted *p* < 0.05. Venn diagrams were constructed using a bioinformatics online tool hosted by WeiShengxin (http://www.bioinformatics.com.cn/).

### Construction of protein–protein interaction (PPI) networks

The identified intersecting genes served as alternative target genes. These genes were imported into the Search Tool for the Retrieval of Interacting Genes/Proteins (STRING) database (https://string-db.org/). The species was set to "*Homo sapiens*" and the confidence level was adjusted to "high confidence (0.4)". Using the Cytoscape software, we constructed a PPI network to analyze the interaction between proteins, aiming to elucidate the biomolecular interaction between associated proteins^11^. To further analyze the PPI network and identify core target genes, we utilized the CytoHubba plugin within Cytoscape, specifically employing clustering analysis^12^. We also ranked the nodes in the network using the degree algorithm, and we identified and visualized the top four core genes.

### Gene ontology (GO) and Kyoto Encyclopedia of Genes and Genomes (KEGG) pathway enrichment analyses

GO and KEGG enrichment analyses were conducted using the database for annotation, visualization, and integrated discovery (DAVID) available at (https://david.ncifcrf.gov/home.jsp). Both analyses used an adjusted *p* < 0.05. The GO analysis classifies enriched terms into three categories: biological process (BP), molecular function (MF), and cellular component (CC), each aimed at describing gene function. KEGG enrichment analysis offered annotation information on signaling pathways, thereby providing a systematic analysis of the potential roles these genes may play within cells^13^. The visualization of the GO and KEGG results was accomplished using the WeiShengxin website.

### Survival analysis and correlation expression of hub genes

To evaluate the prognostic significance of hub genes in GBM, we performed survival analyses, specifically examining overall survival (OS) and disease-free survival (DFS). The RNA sequencing data and corresponding clinical data of patients with GBM were obtained from the GEPIA2 dataset. Additionally, we used the Chinese Glioblastoma Genome Atlas platform (http://www.cgga.org.cn/) to analyze the correlation among the hub genes in GBM. This platform provides mRNA sequencing datasets for brain tumors paired with clinical information. Furthermore, we employed the Tumor Immune Estimation Resource (TIMER) 2.0 (http://timer.comp-genomics.org/timer/) to explore the correlations between hub genes and tumor-infiltrating immune cells (B cell, CD8^+^T cell, CD4^+^T cell, myeloid dendritic cell, macrophage, and neutrophil).

### Validation analysis of hub genes

TIMER 2.0 was used to examine the expression differences of hub genes between normal tissues and various cancer types. The differential expression of these genes in normal tissues was evaluated using the box diagram available in GEPIA2 and compared with the different expression levels across various GBM tissues. Furthermore, the Human Protein Atlas (HPA) database (https://www.proteinatlas.org/) was used to examine the protein expression, distribution, and subcellular localization of hub genes through immunohistochemistry in both normal tissues and GBM samples.

### Molecular docking analysis

The three-dimensional structure of BCA (MOL003341) was obtained from PubChem. Candidate protein crystal structures were downloaded from the Protein Data Bank (https://www.rcsb.org/) database. Docking calculations were performed using DockThor^14^ (https://dockthor.lncc.br/v2/) to predict the stable binding conformation and identify molecular interactions between the receptor and ligand. The results of the molecular docking between BCA and the target proteins were visualized using PyMol (version 2.0.6).

### Reagents

Cell Counting Kit-8 (CCK-8) was purchased from ABMole BioScience (Shanghai, China). Fetal bovine serum (FBS) was obtained from Sangon Biotech (Shanghai, China) Co., Ltd. p-AKT was purchased from Cell Signaling Technology (Danvers, MA, USA). Antibodies against AKT, EGFR, MMP‐9, caspase3, cleaved-caspase3, and β-actin were obtained from Proteintech Group (Wuhan, China). HRP-conjugated affinity-purified goat anti-mouse IgG and goat anti-rabbit IgG were also obtained from Proteintech Group (Wuhan, China).

### Cell line and culture

The human GBM cell line U251 was purchased from the Biological Cell Bank of the China Academy of Sciences (Shanghai, China) and maintained in our laboratory at Lanzhou University Second Hospital. Cells were cultured in Dulbecco’s modified Eagle’s medium (DMEM)/high‐glucose medium supplemented with 10% FBS and 1% penicillin‒streptomycin. All cells were incubated in a 5% CO_2_ environment at 37 °C.

### Cell viability analysis

Cell viability was determined using the CCK-8 assay. Cells were seeded in 96-well plates at a density of 5 × 10^3^ cells/well. Various concentrations of BCA (10, 20, 40, 80, 100, and 200 μM) were sequentially added to each well for 24 and 48 h. Subsequently, 10 µL of CCK-8 solution was added to each well and incubated for an additional 3 h. The half-maximal inhibitory concentration (IC50) of BCA on the U251 cells was determined using GraphPad Prism 8.0.

### Apoptosis and cell cycle analyses

The Annexin V-APC/7-AAD detection kit was used to evaluate the apoptosis of GBM U251 cells induced by BCA. Cells were seeded in six-well plates at a density of 5 × 10^5^ cells/well. After the cells adhered to the wall, they were treated with different concentrations of BCA (0, 50, and 100 μM) for 48 h. According to the manufacturer's instructions, cells with supernatant were harvested and washed twice with ice-cold phosphate-buffered saline. Subsequently, for apoptosis analysis, cells were incubated in 5 μL of Annexin V-APC solution and 10 μL of 7-AAD solution at room temperature for 5 min. Cells were collected and fixed with ice‐cold 70% ethanol at 4 °C overnight. The cells were then stained with propidium iodide and RNase for cell cycle analysis; subsequently, they were analyzed using a flow cytometer. The visualization of the staining data was performed using FlowJo 10.0.

### Western blotting

After the treated cells were harvested, the total protein was extracted using RIPA. The proteins were separated using SDS‐PAGE. After electrophoresis, they were transferred onto PVDF membranes and blocked using 5% non‐fat milk. Thereafter, membranes were cut and washed with TBST then incubated with primary antibodies. After three TBST washes, membranes were incubated with the corresponding HRP-linked secondary antibody for 1 h. The membranes were exposed to enhanced chemiluminescence (ECL) reagent. The density of bands was measured using an image analysis system and quantified as the gray ratio with the ImageJ software^15^.

### Statistical analysis

All experiments were performed a minimum of three times. All the data were statistically analyzed using GraphPad Prism software (version 8.0). Data are presented as mean ± standard deviation (SD) and evaluated using one-way analysis of variance, followed by the Tukey’s multiple comparisons post-hoc test. Statistical significance was set at *p* < 0.05.

## Results

### Therapeutic targets for BCA against GBM

**Figure 2:**
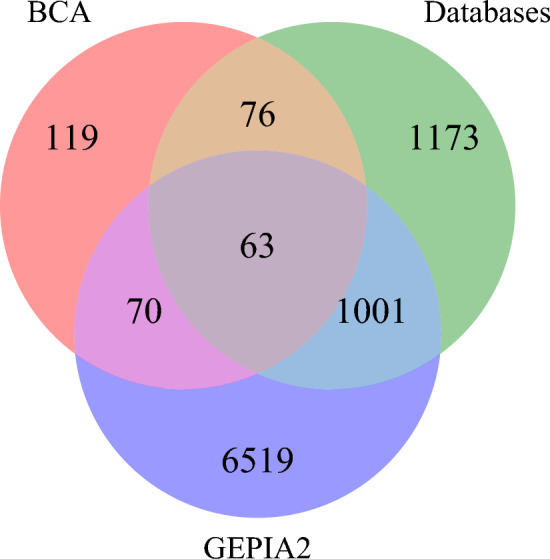
The Venn diagrams of BCA and GBM targets. The diagram illustrates BCA targets in red, GBM targets collected from three disease databases (DisGeNet, GeneCards and TTD) in green, and DEGs of GBM from the GEPIA2 database in blue.

A total of 328 BCA target genes were sourced from the Swiss Target Prediction and PharmMapper databases. Additionally, 5,654 GBM disease target genes were compiled by integrating data from DisGeNet, GeneCards, and TTD. From the GEPIA2 database, 7664 common DEGs, which constituted 5225 upregulated genes and 2439 downregulated genes, were identified. To further investigate the mechanism of BCA treatment for GBM, 63 intersecting genes were identified and designated as core target genes (Fig. 2).

### PPI network of BCA and GBM

**Figure 3:**
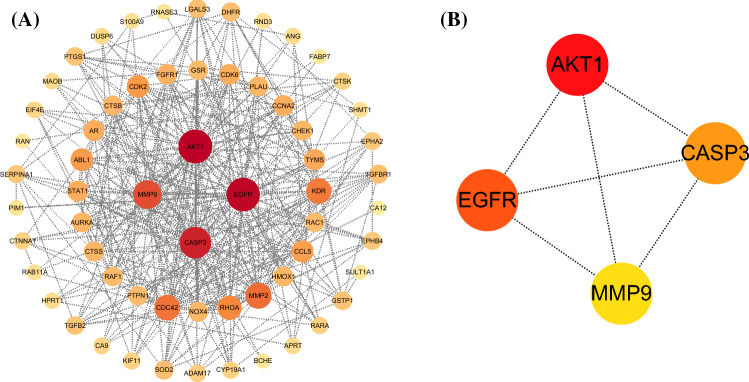
PPI network of BCA targets against GBM. (**A**) The PPI network of BCA- and GBM-related targets. (**B**) The top four hub genes of the PPI network using the CytoHubba algorithm within Cytoscape.

PPI networks are valuable tools for systematically investigating the molecular mechanisms and drug targets of complexes. We used the STRING database to construct a PPI network of BCA- and GBM-related targets (Fig. 3A). Cytoscape software was used to visualize the PPI network diagram. Subsequently, using degree topological analysis methods, four major targets were identified by the CytoHubba plugin, namely, *AKT1*, *EGFR*, *CASP3*, and *MMP9* (Fig. 3B).

### Hub target genes in DEGs

**Figure 4:**
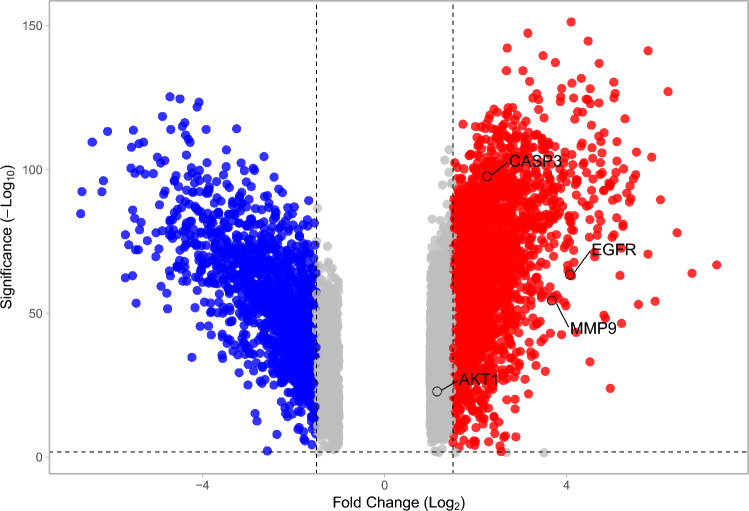
DEGs in GBM; *AKT1*, *EGFR*, *CASP3*, and *MMP9* were upregulated in the GBM group.

DEGs in GBM were sourced from the GEPIA2 database and analyzed using VolcaNoseR. Four critical target genes—*AKT1*, *EGFR*, *CASP3*, and *MMP9*—were identified and are highlighted in a volcano plot (Fig. 4). These genes were upregulated in the GBM group.

### GO and KEGG pathway enrichment analysis

**Figure 5:**
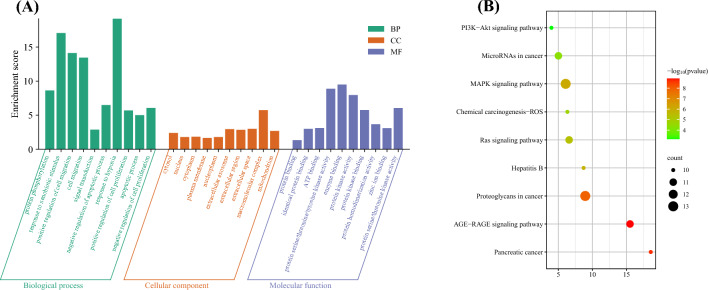
GO and KEGG analyses show the anti-tumor effects of BCA on biological processes. (**A**) GO enrichment analysis on screened genes. (**B**) KEGG functional enrichment analysis of common targets.

GO enrichment analysis of the 63 target genes using the DAVID database revealed the anti-tumor effects of BCA on biological processes. Using a screening criterion of *p* < 0.05, 443 BP, 66 CC, and 116 MF terms were identified. The results indicated that the primary BP terms were associated with signal transduction, protein phosphorylation, apoptotic processes, and cell migration. The CC terms primarily encompassed the cytosol, nucleus, cytoplasm, and plasma membranes. MF was linked to various molecular activities, including protein binding, ATP binding, zinc ion binding, and protein serine/threonine/tyrosine kinase activity (Fig. 5A). As depicted in Fig. 5B, 147 KEGG enrichment analysis signaling pathways were identified, with the top 10 significant pathways (*p* < 0.05) being the PI3K-Akt, Ras, and MAPK signaling pathways. Among these, the PI3K-Akt signaling pathway emerged as the most enriched, suggesting that BCA likely exerts its therapeutic effects on GBM via these pathways.

### Survival analysis and hub gene correlations

**Figure 6:**
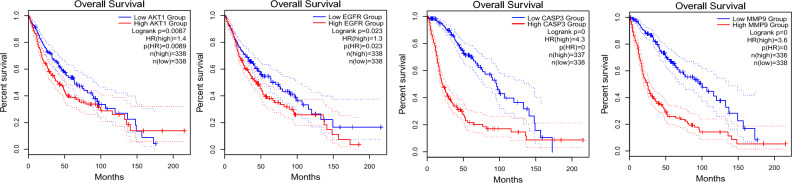
Survival analysis using the hub genes—overall survival (OS).

**Figure 7:**
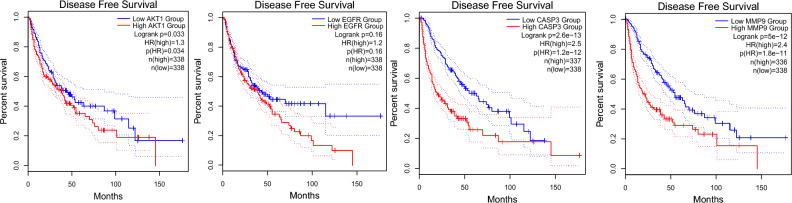
Survival analysis using the hub genes—disease free survival (DFS).

**Figure 8:**
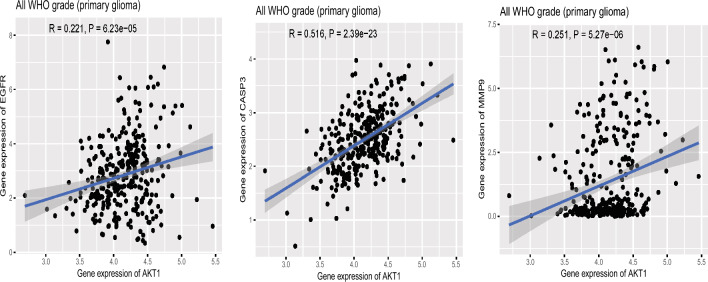
Positive correlation between AKT1 and the expression of EGFR, CASP3, and MMP9 proteins.

To clarify the impact of hub genes on the survival and prognosis of patients with GBM, a survival analysis was conducted (*p* < 0.05; Figs. 6, 7). Certain hub genes like *AKT1*, *EGFR*, *CASP3*, and *MMP9* were positively associated with the survival of patients with GBM. To further investigate the underlying mechanisms of hub genes in tumorigenesis, the expression level of AKT1 was assessed. It was positively correlated with those of EGFR (r = 0.221,* p* = 6.23 × 10^–5^), CASP3 (r = 0.516, *p* = 2.39 × 10^–23^), and MMP9 (r = 0.251, *p* = 5.27 × 10^–6^) (Fig. 8).

### Differential expression of the hub genes in normal and GBM tissues

**Figure 9:**
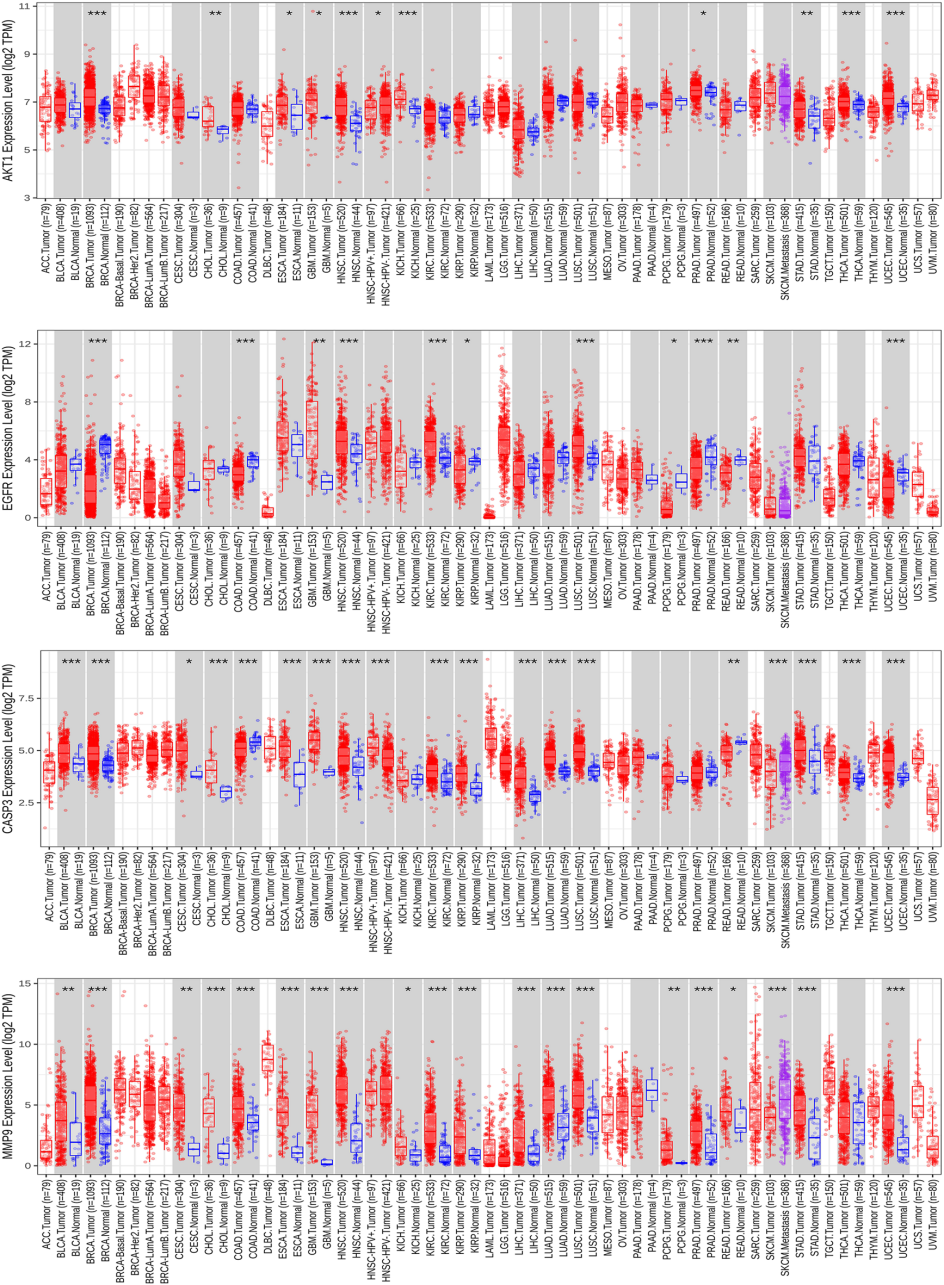
Expression of hub genes in normal and multiple tumor types (*p < 0.05, **p < 0.01, ***p < 0.001).

**Figure 10:**
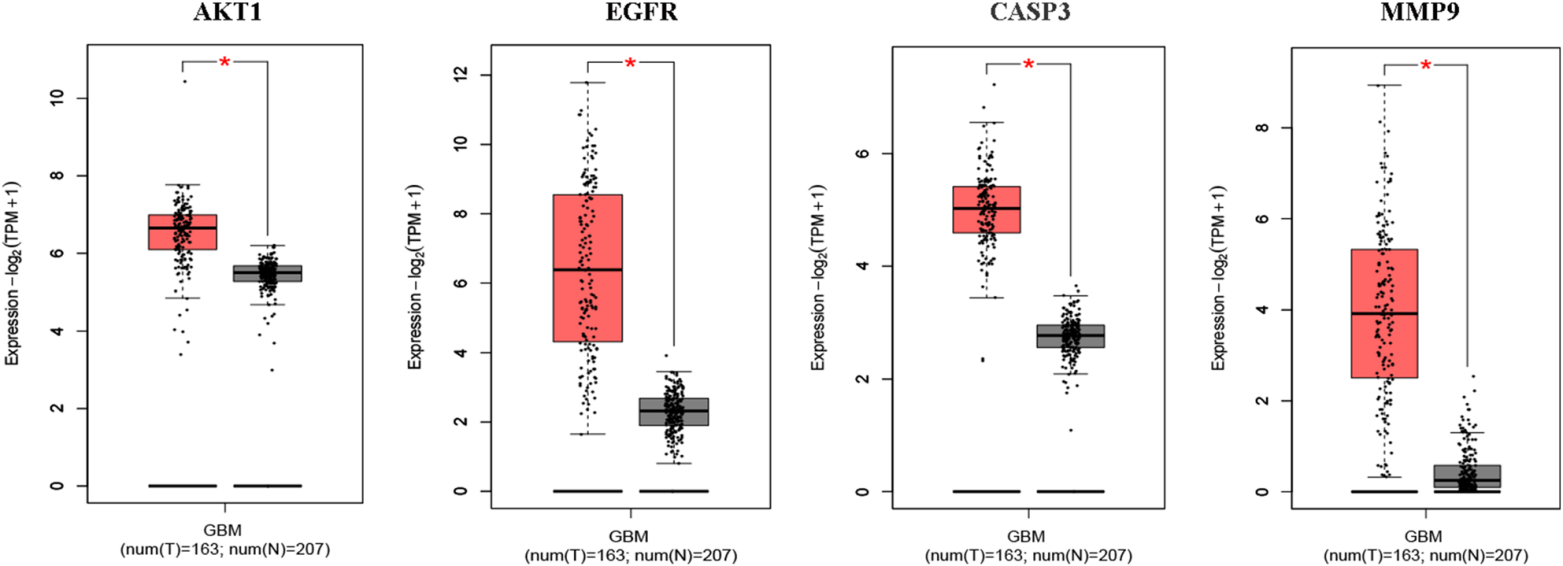
Expression of hub genes in normal tissue and GBM as per TCGA database (Box Plot).

**Figure 11:**
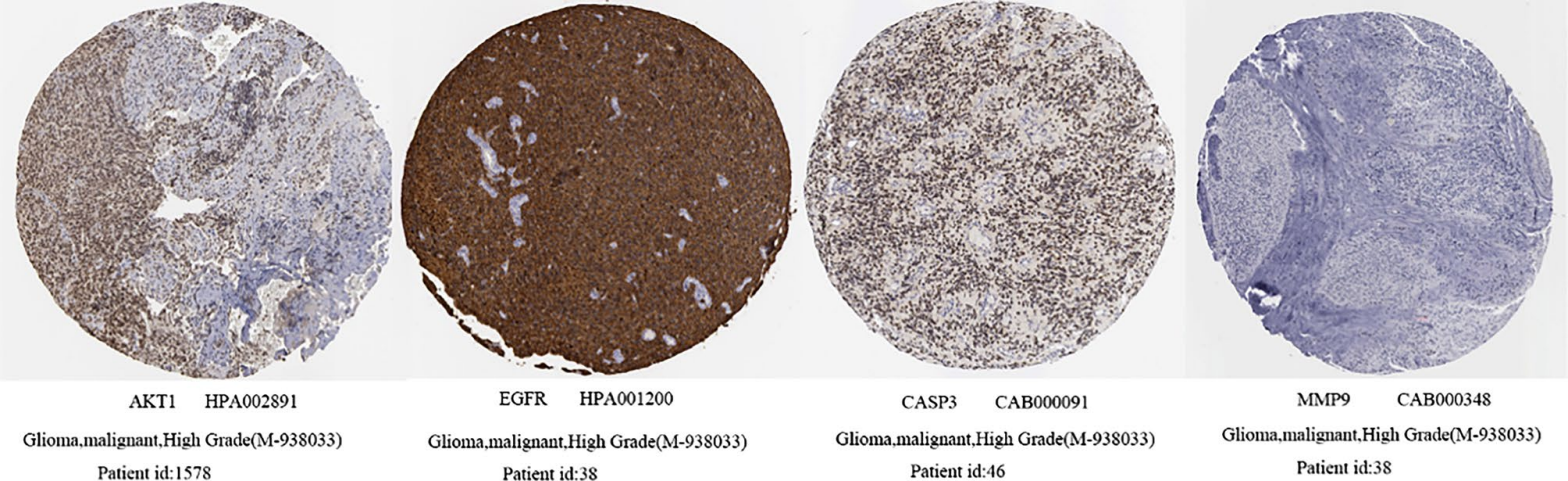
Detection of AKT1, EGFR, CASP3, and MMP9 proteins using immunohistochemistry from the HPA database.

First, we examined the expression levels of differentially expressed hub genes across multiple tumor types using pan-cancer analysis in the TIMER 2.0 database. This analysis indicated that the hub genes *AKT1*, *EGFR*, *CASP3*, and *MMP9* were significantly overexpressed in GBM tissues (*p* < 0.01), while maintaining low expression levels in normal tissues (Fig. 9). Similarly, mRNA level differences between GBM and normal tissues were evident in the box plots from the GEPIA2 database, highlighting *AKT1*, *EGFR*, *CASP3*, and *MMP9* (Fig. 10). Finally, immunohistochemical staining data from the HPA database confirmed higher protein levels of AKT1, EGFR, CASP3, and MMP9 in GBM tissues compared to those of normal tissues, aligning with the mRNA findings (Fig. 11).

### Tumor-infiltrating immune cells

We also examined the correlation between the expression of hub genes and levels of tumor-infiltrating immune cells in GBM. The expression levels of AKT1 (r = 0.311; *p* = 2.06 × 10^–4^), EGFR (r = 0.288; *p* = 6.15 × 10^–4^), and CASP3 (r = 0.232; *p* = 6.24 × 10^–3^) were significantly positively correlated with purity, whereas only MMP9 showed a significant negative correlation with purity (r = − 0.164; *P* = 5.46 × 10^–2^). These results suggest that the expression levels of the hub genes are associated with the infiltration levels of immune cells and play an important role in modulating GBM immune function (Table 1).

**Table 1:** Correlation between the target proteins and tumor-infiltrating immune cells.

Gene name	Parameters	Purity	B cell	CD8^+^	CD4^+^	Macrophage	Neutrophil	Dendritic cell
AKT1	R	0.311	0.013	− 0.318	0.187	− 0.008	− 0.127	0.134
	*P* value	0.000206	0.878	0.000153	0.0287	0.924	0.14	0.119
EGFR	R	0.288	0.07	− 0.142	0.195	− 0.04	0.005	0.041
	*P* value	0.000615	0.417	0.0975	0.0227	0.639	0.957	0.63
CASP3	R	0.232	− 0.181	0.033	− 0.103	− 0.163	0.009	0.027
	*P* value	0.00624	0.0344	0.705	0.231	0.0563	0.917	0.751
MMP9	R	− 0.164	− 0.004	0.128	− 0.263	0.019	0.06	0.147
	*P* value	0.0546	0.961	0.135	0.0019	0.822	0.484	0.0858

### Molecular docking analysis

**Figure 12:**
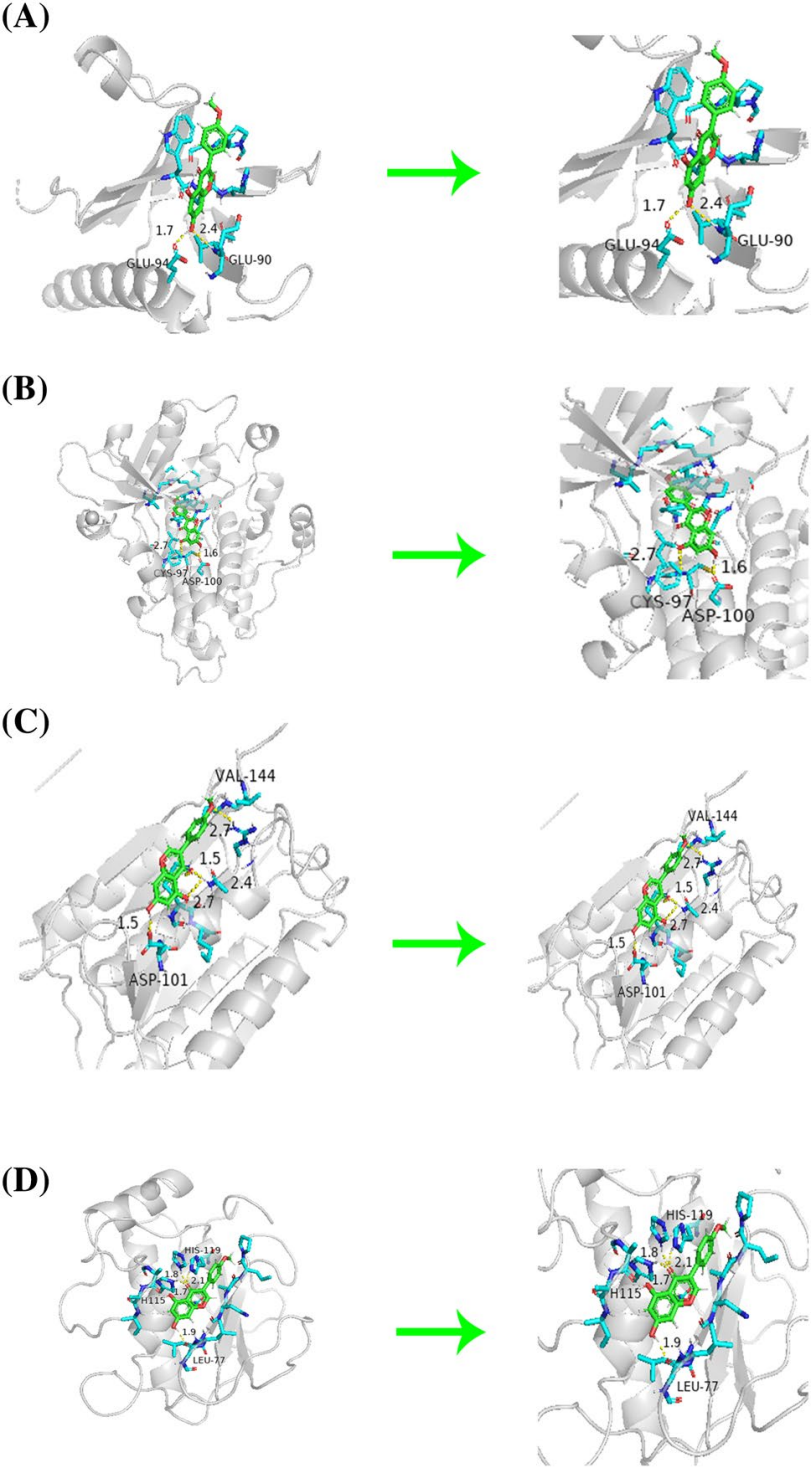
The Molecular Docking maps of BCA and AKT1 (**A**), EGFR (**B**), CASP3 (**C**), and MMP9 (**D**).

We performed molecular docking of four hub genes—*AKT1*, *EGFR*, *CASP3*, and *MMP9*—with BCA using DockThor. The molecular docking images were visualized using the PyMol software, and the results are presented in Fig. 12. The binding activities of the large molecule receptor proteins and small molecule ligands were identified and evaluated using the docking energy between them. The lower the binding energy, the greater the binding force. Notably, molecular docking studies confirmed the interactions between BCA and key target proteins, providing primary evidence that BCA inhibits GBM by targeting these key target proteins.

### BCA inhibited GBM cell viability

**Figure 13:**
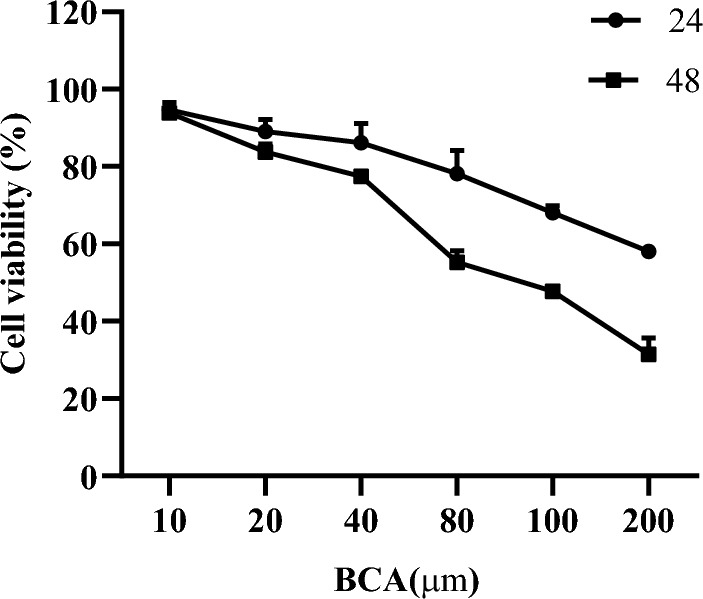
Viability of U251 cells treated with BCA. Viability of BCA-treated cells at different concentrations (10, 20, 40, 80, 100, and 200 µM) for 24 and 48 h. The data are presented as mean ± SD of three independent experiments.

CCK-8 assays were performed to investigate the effect of BCA on the cell viability of GBM U251 cells. The cell viability assay revealed dose- and time-dependent inhibition of cell proliferation induced by BCA. The viability of U251 cells significantly decreased, with an observed IC50 value of 98.37 ± 2.21 μM at 48 h. Therefore, we selected 50 and 100 μM concentrations for subsequent experiments (Fig. 13).

### BCA-induced apoptosis and cell cycle arrest in U251 cells

**Figure 14:**
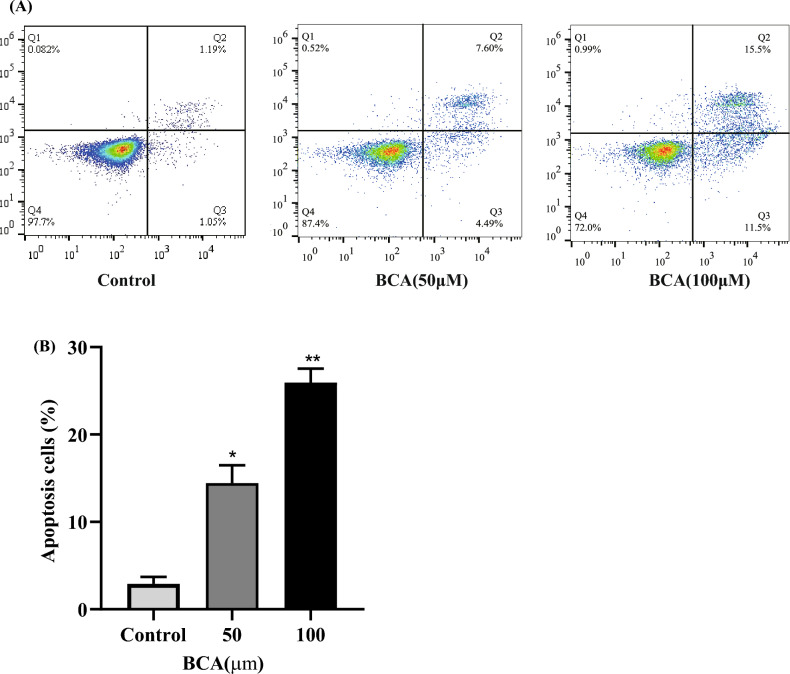
BCA-induced apoptosis of U251 cells. (**A**) The impact of BCA at different concentrations over 48 h on apoptosis in U251 cells was determined using flow cytometry. (**B**) The total ratio of apoptotic cells was calculated. Data are expressed as mean ± SD from three independent experiments; * *p* < 0.05 and ** *p* < 0.01 versus control.

To further analyze the effect of apoptosis in U251 cells by BCA, an Annexin V-APC/7-AAD double staining assay was performed. The percentage of apoptotic cells was significantly higher in the BCA groups than in the control group. Apoptosis cells increased from 2.90 ± 0.81 to 25.93 ± 1.61%. The above results confirmed that BCA could inhibit the proliferation of U251 cells by inducing apoptosis (Fig. 14).

**Figure 15:**
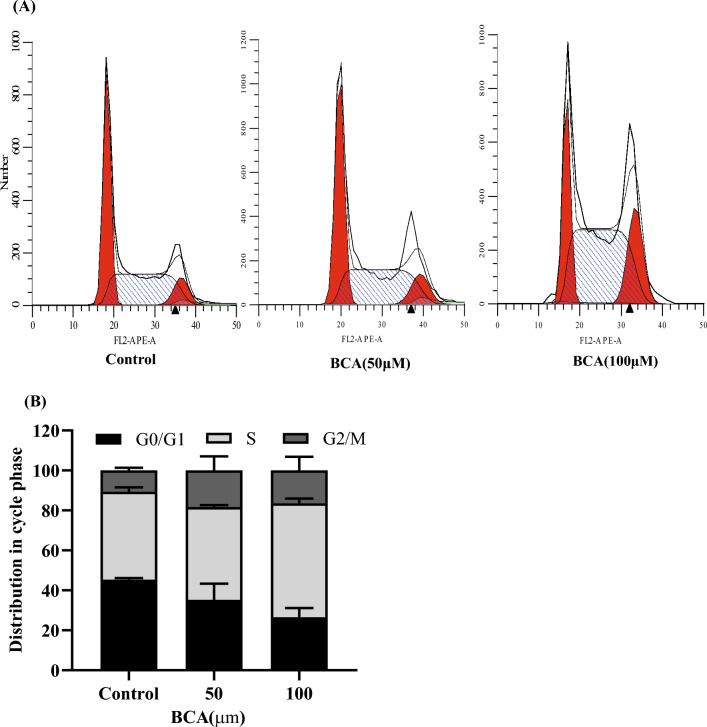
Effects of BCA on the cell cycle of U251 cells as evaluated using flow cytometry. (**A**) The cell cycle distribution of U251 cells treated with BCA at different concentrations. (**B**) The histogram shows the distribution of cells (%) in the G0/G1, S, and G2/M phases.

DNA flow cytometry was used to evaluate the effect of BCA on the cell cycle, significantly exploring its molecular mechanisms. The cell cycle distribution underwent significant changes in the S phase arrest in BCA-treated cells compared to those in the control group. The percentage of S phase cells was 44.02 ± 2.2% in the control group and 56.96 ± 2.43% after U251 cells were treated with 100 μM BCA, showing an increased percentage of S phase cells compared with control group cells (Fig. 15).

### BCA regulated the expression of core proteins in U251 cells

**Figure 16:**
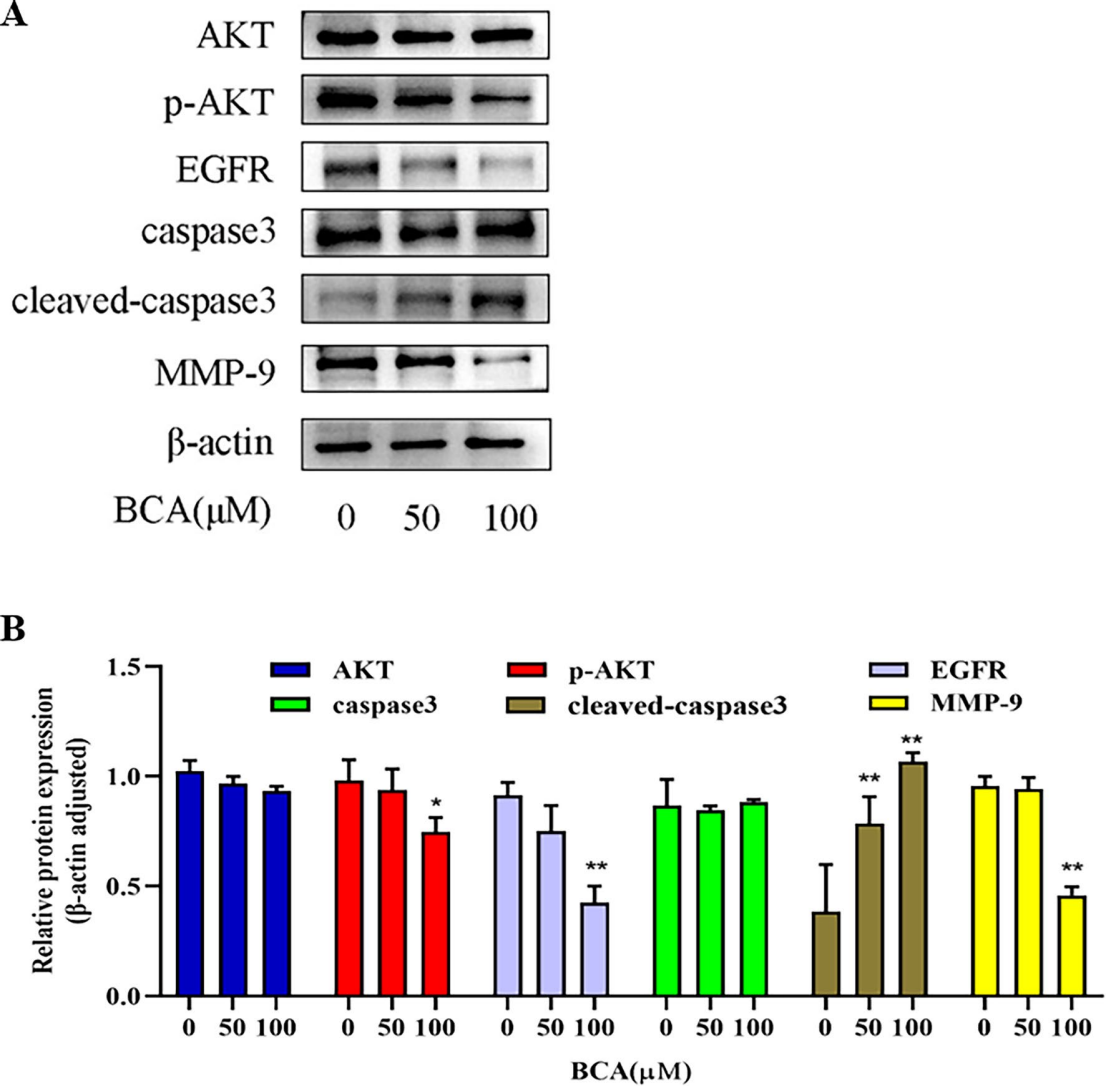
Effects of BCA on core protein expression levels in U251 cells. (**A**) The expression levels of AKT1, p-AKT1, EGFR, CASP3, cleaved-caspase3, and MMP9 proteins were detected using western blotting, and the relative protein levels were determined using the ImageJ software. (**B**) Data are shown as mean ± SD from three independent experiments. β‐actin served as the control for determining protein expression levels. *p < 0.05 and **p < 0.01 versus control.

To further evaluate the effects of BCA, the expression of core proteins was analyzed using western blotting. BCA decreased the expression of p-Akt, EGFR, and MMP9 dose-dependently compared with those seen in the control group. However, the expression levels of AKT and caspase-3 proteins in the experimental group showed no significant changes. Additionally, BCA increased the expression levels of the cleaved-caspase3 protein (Fig. 16). Western blot graphs analyses are provided in the Supplementary Materials (Supplementary File S1).

## Discussion

Currently, the prediction of potential relationships between drugs and diseases primarily relies on expensive and time-consuming biological experiments^16^. Therefore, potential drugs must be predicted using the bioinformatics method. Research in computational biology for interaction prediction could provide valuable insights into genetic markers and their association with diseases, thereby advancing bioinformatics methods as a cost-effective and robust solution^17,18^. Machine learning, used in various fields such as cancer risk assessment^19^, carcinogenicity prediction^20^, quality of life and survival prediction^21^, miRNA-lncRNA interaction prediction^22–24^ and metabolite-disease associations^16,25^ has also advanced prediction techniques, aiding in the early stages of drug development by prioritizing molecules^20,26^.

In this study, we performed an integrated and comprehensive bioinformatics analysis to identify reliable target genes for GBM. Initially, we identified BCA targets using online pharmacology databases. Subsequently, we screened GBM-expressed genes from DisGeNet, GeneCards, and TTD, identified DEGs using GEPIA2, and analyzed them using VolcaNoseR. By integrating data from drug, disease, and DEG interaction networks, we identified 63 crossover target genes for GBM treatment. PPI network analysis revealed *AKT1*, *EGFR*, *CASP3*, and *MMP9* as highly relevant to treatment genes. These genes are closely associated with GBM occurrence, development, and prognosis. GO enrichment analysis showed that these targets were involved in processes such as cell migration, proliferation, apoptosis, and other multicellular biological processes crucial for GBM onset and progression. These genes were associated with protein binding and protein serine/tyrosine/ threonine kinase activity in the MF category. KEGG pathway enrichment analysis revealed their participation in the PI3K-Akt and MAPK signaling pathways. Additionally, molecular docking analysis confirmed the feasibility of the binding modes, demonstrating that BCA had a high affinity for these four key target genes.

Subsequently, we systematically researched the biological functions and potential regulatory pathways of hub genes in GBM. We aimed to assess the expression and prognostic value of AKT1, EGFR, CASP3, and MMP9 in GBM. We found that these hub genes were notably upregulated in GBM samples and correlated with tumor grade. High expression levels were closely linked to poor OS and DFS in patients with GBM. Additionally, we observed a positive correlation between AKT1 and EGFR, CASP3, and MMP9 expression. These results strongly suggest the significance of these four genes in prognosis and their potential as hub genes in GBM treatment using BCA.

AKT1 is a serine/threonine protein kinase that modulates protein synthesis and transcription. While the expression of AKT1 in the GBM group was not more than double that in the control group, the difference between the two groups was statistically significant. Growing evidence indicates that AKT1 plays a key role in the progression and aggressiveness of GBM, affecting cell proliferation, apoptosis, and migration. Elevated AKT1 expression has been observed in various cancer types, including GBM, during disease progression. Consequently, AKT1 is viewed as a critical factor in tumor advancement^27^. Inhibiting the Akt/mTOR signaling pathway triggers both autophagy and apoptosis in glioma cells^28^.

EGFR, a transmembrane glycoprotein and a member of the ErbB family of receptor tyrosine kinases, is frequently overexpressed and mutated in GBM and many other cancers. Its presence enhances GBM proliferation, invasion, and drug resistance^29^. EGFR is considered a promising candidate target in advanced precision medicine for patients with central nervous system tumors^30^. A study by Zhou et al.^8^ has indicated that inhibiting the EGFR/SRC/STAT3 signaling pathway suppresses cell proliferation, induces apoptosis, and blocks the cell cycle in the G2/M phase of gliomas. Recent research demonstrates that blocking EGFR signaling using the EGFR inhibitor AG1478 increases the sensitivity of glioma cells to temozolomide^31^.

CASP3 is activated during apoptosis upon cellular exposure to drugs and radiotherapy and is often considered a marker of cancer treatment efficacy^32^. Recent studies have revealed that CASP3 also plays non-apoptotic roles, such as promoting tumor migration, invasion, and relapse. CASP3 interacts with DNA, stimulating angiogenesis by inducing proangiogenic gene expression and activating pathways that promote endothelial cell activation, tumor recurrence, and chemotherapy resistance^33^. Tumor cells with CASP3 knockout are highly sensitive to radiotherapy and chemotherapy due to the inhibited epithelial-mesenchymal transition^34^.

MMP9 was notably overexpressed among the DEGs identified in GBM, according to TCGA database. MMP9 plays a critical role in extracellular matrix degradation, promoting tumor tissue invasion and metastasis^35^. Elevated MMP9 levels show a positive correlation with GBM progression, indicating a poor prognosis when found at high levels in brain tumor tissues^36^. Blue light-activated curcumin induces apoptosis in GBM by downregulating MMP pathways in a ROS-dependent manner^37^.

In our study, we explored the potential link between immune regulation and hub genes and found that tumor-infiltrating immune cells play a significant role in prognosis-related molecular mechanisms, thereby promoting tumor progression and therapeutic resistance. We also found that BCA hindered proliferation, induced cell apoptosis, and caused cell cycle arrest in a concentration-dependent manner. Our findings indicated that BCA promotes GBM cell apoptosis by upregulating cleaved caspase-3 expression and inhibits GBM progression by downregulating p-AKT, EGFR, and MMP9 expression. These results shed new light on the diverse protein functions affected by BCA against GBM, offering insights to guide the development of viable disease control strategies^38^.

This study had certain limitations that require thorough investigation in future research owing to the complexity of the effects of BCA on GBM. Further in vivo experiments are required to explore the anti-GBM effects of BCA. In addition, confirming the therapeutic effect of BCA for treating patients with GBM is imperative. Future studies should determine the potential of BCA as an adjuvant therapy to enhance the effectiveness of chemotherapeutic agents.

## Conclusion

We examined the molecular mechanisms underlying the impact of BCA on GBM using an integrative bioinformatics approach with in vitro experiments. This study lays a theoretical foundation for further research and development of novel GBM therapies.

### Supplementary Information

Supplementary Information 1.

Supplementary Information 2.

Supplementary Information 3.

Supplementary Information 4.

Supplementary Information 5.

Supplementary Information 6.

Supplementary Information 7.

## Data Availability

The datasets used and/or analysed during this study are available from the corresponding author on reasonable request.
