# Supplementary figures and images for: Exploring the therapeutic mechanisms and prognostic targets of Biochanin A in glioblastoma via integrated computational analysis and in vitro experiments
Source: Sci Rep. 2024 Feb 15;14:3783. doi: 10.1038/s41598-024-53442-0 (PMC10869694; doi:10.1038/s41598-024-53442-0)

AKT1 65kDa

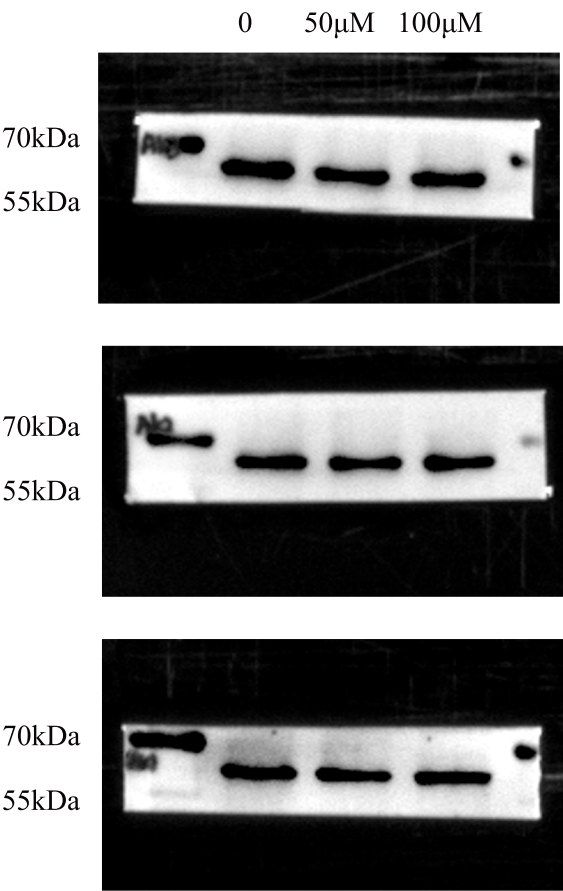

Supplement: Supplementary file 1 — Supplementary Information 1. [file 41598_2024_53442_MOESM1_ESM.pdf]

caspase3 32kDa

0 50μM 100μM

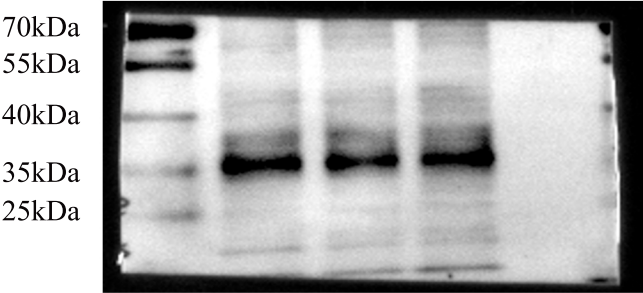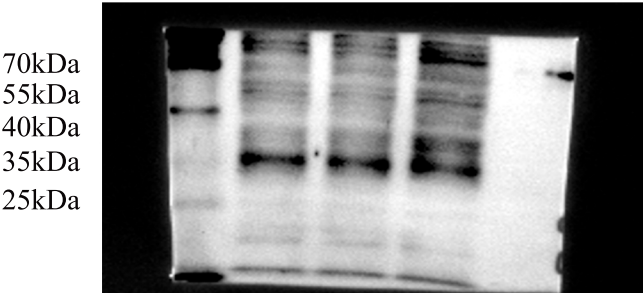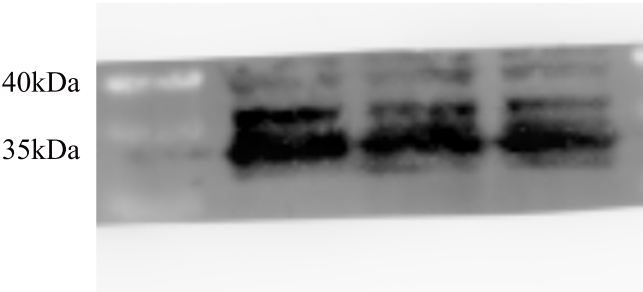

Supplement: Supplementary file 2 — Supplementary Information 2. [file 41598_2024_53442_MOESM2_ESM.pdf]

cleaved-caspase3 20kDa

0 50μM 100μM

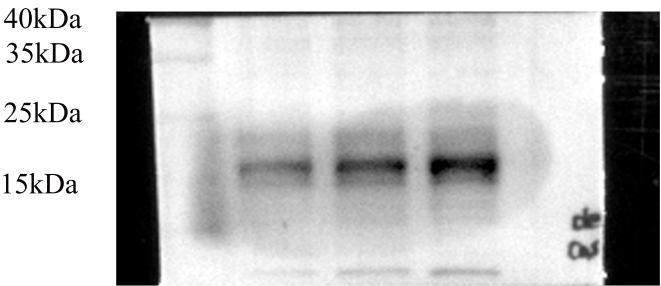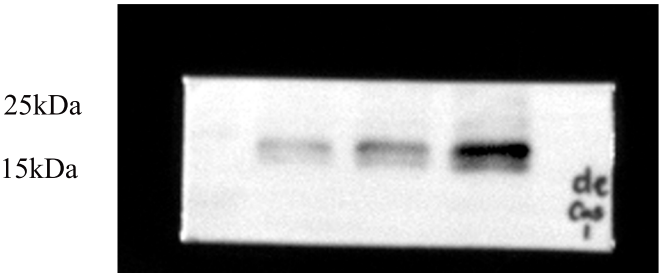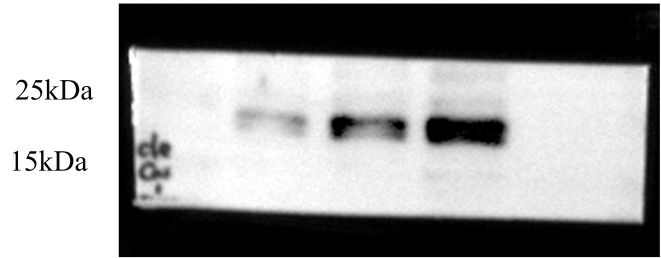

Supplement: Supplementary file 3 — Supplementary Information 3. [file 41598_2024_53442_MOESM3_ESM.pdf]

EGFR 150kDa

0    50μM    100μM

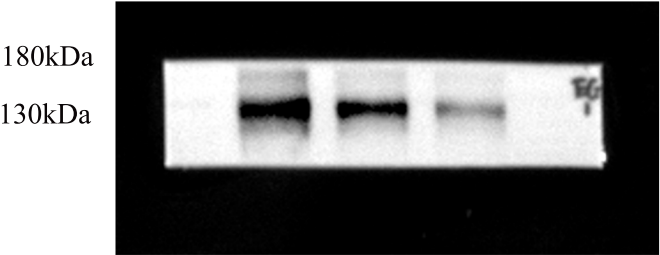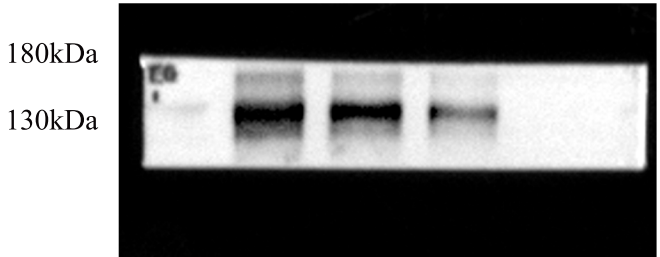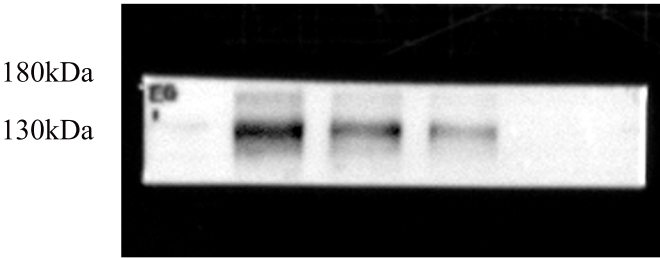

Supplement: Supplementary file 4 — Supplementary Information 4. [file 41598_2024_53442_MOESM4_ESM.pdf]

$\beta$ -actin 42kDa

0 50  $\mu$ M 100 $\mu$ M

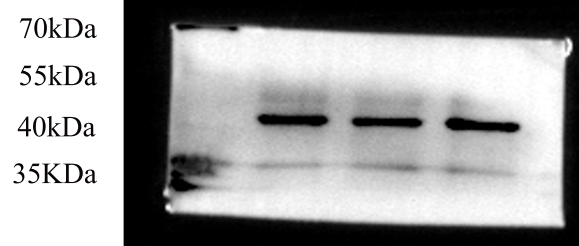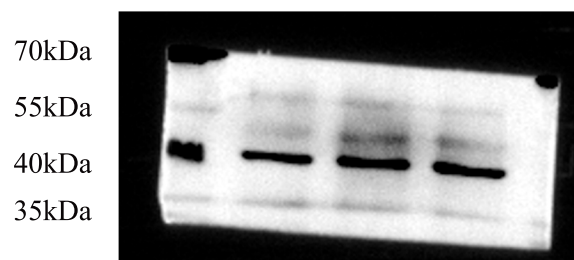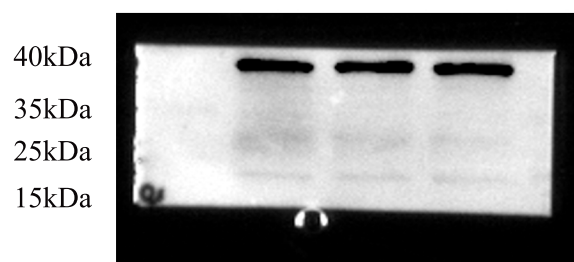

Supplement: Supplementary file 5 — Supplementary Information 5. [file 41598_2024_53442_MOESM5_ESM.pdf]

MMP-9 110kDa

0 50μM 100μM

130kDa  
100kDa

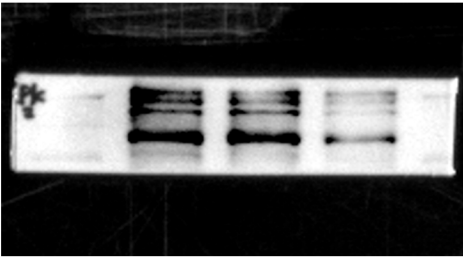

130kDa  
100kDa

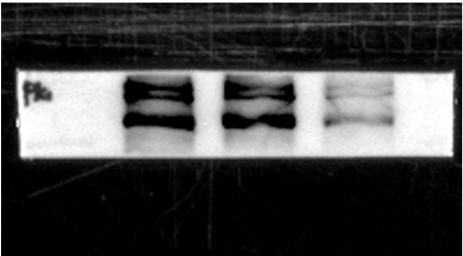

130kDa  
100kDa

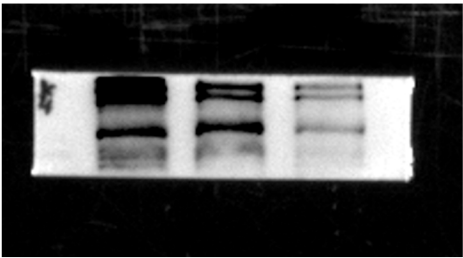

Supplement: Supplementary file 6 — Supplementary Information 6. [file 41598_2024_53442_MOESM6_ESM.pdf]

p-AKT 65kDa

0 50μM 100μM

70kDa  
55kDa

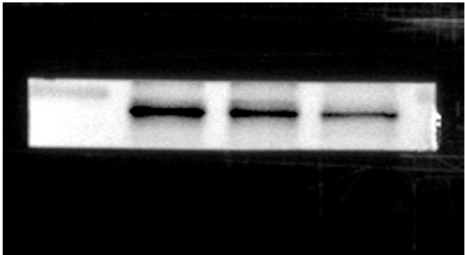

70kDa  
55kDa

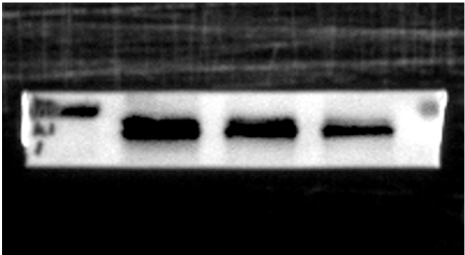

70kDa  
55kDa

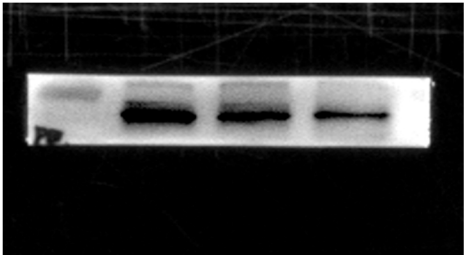

Supplement: Supplementary file 7 — Supplementary Information 7. [file 41598_2024_53442_MOESM7_ESM.pdf]
